# Supplementary figures and images for: SSD1 suppresses phenotypes induced by the lack of Elongator-dependent tRNA modifications
Source: PLoS Genet. 2019 Aug 29;15(8):e1008117. doi: 10.1371/journal.pgen.1008117 (PMC6738719; doi:10.1371/journal.pgen.1008117)

A

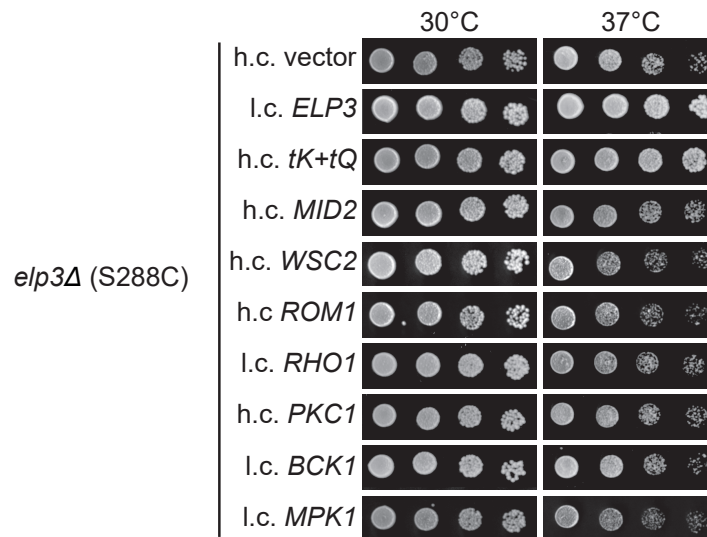

B

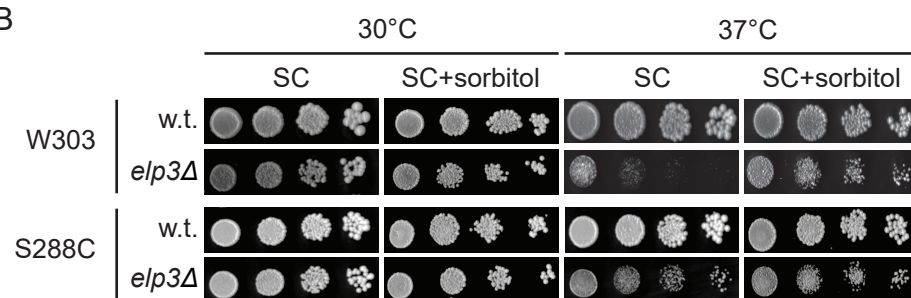

Supplement: S1 Fig — (A) Growth of the elp3Δ (MJY1036) strain carrying the indicated high-copy (h.c.) or low-copy (l.c.) LEU2 plasmids. Cells were grown over-night at 30°C in liquid SC-leu medium, serially diluted, spotted on SC-leu plates, and incubated at 30°C or 37°C for 3 days. (B) The wild-type (W303-1A and BY4741) and elp3Δ (UMY3269 and MJY1036) strains were grown over-night at 30°C in liquid SC medium, serially diluted, and spotted on SC plates and SC plates supplemented with 1M sorbitol. The plates were incubated for 3 days at 30°C or 37°C. (PDF) [file pgen.1008117.s001.pdf]

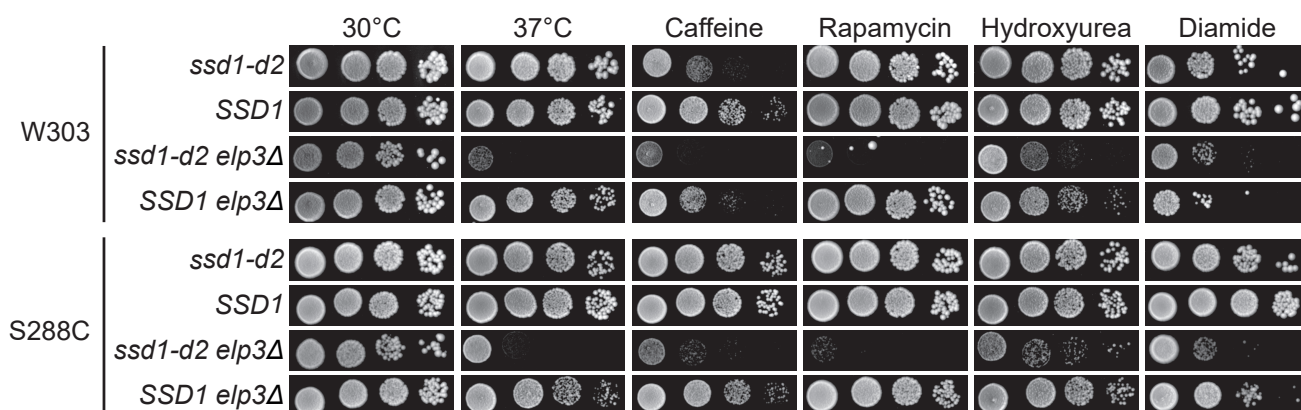

Supplement: S2 Fig — The ssd1-d2 (W303-1B and UMY4433), SSD1 (UMY3386 and BY4742), ssd1-d2 elp3Δ (UMY2843 and UMY4438) and SSD1 elp3Δ (UMY4457 and MJY1037) strains were grown over-night at 30°C in liquid SC medium, serially diluted, and spotted on SC plates and SC plates supplemented with caffeine, rapamycin, hydroxyurea, or diamide. The plates were incubated at 30°C or 37°C for 3 days. (PDF) [file pgen.1008117.s002.pdf]

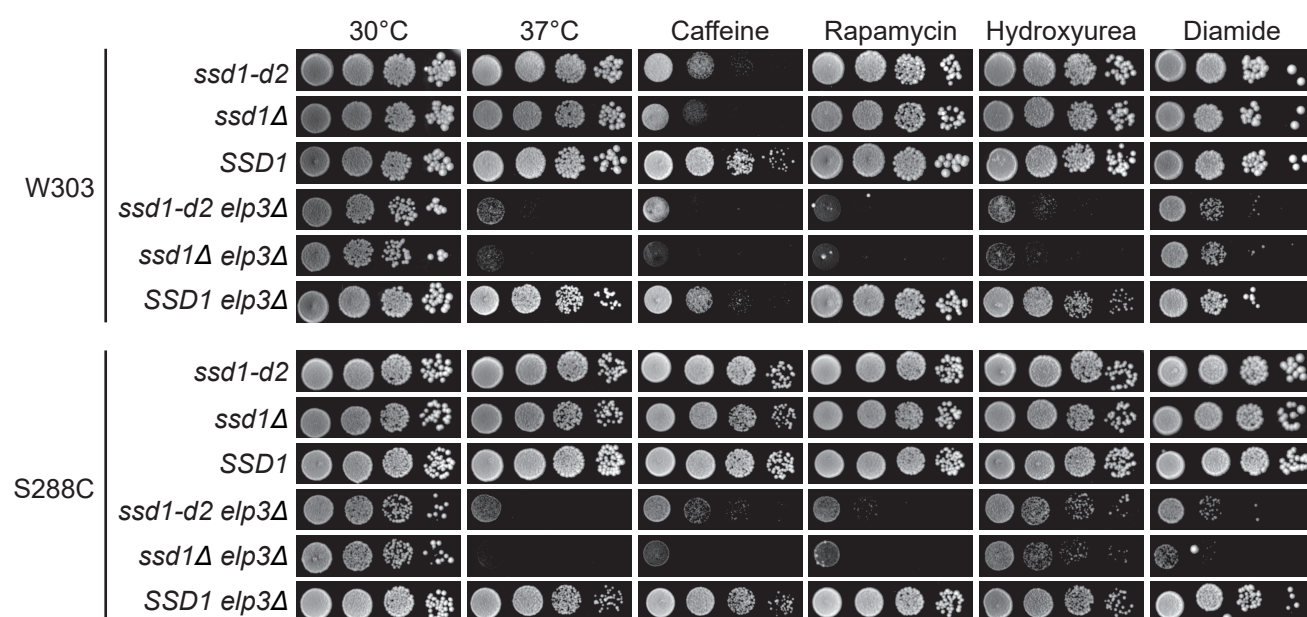

Supplement: S3 Fig — The ssd1-d2 (W303-1A and UMY4432), ssd1Δ (UMY4558 and UMY4559), SSD1 (UMY3385 and BY4741), ssd1-d2 elp3Δ (UMY3269 and UMY4439), ssd1Δ elp3Δ (UMY4574 and MJY1227), and SSD1 elp3Δ (UMY4456 and MJY1036) strains were grown over-night at 30°C in liquid SC medium, serially diluted, and spotted on SC plates and SC plates supplemented with caffeine, rapamycin, hydroxyurea, or diamide. The plates were incubated for 3 days at 30°C or 37°C. (PDF) [file pgen.1008117.s003.pdf]

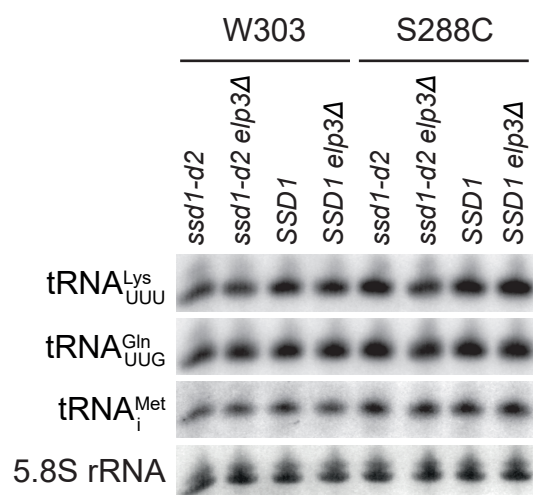

Supplement: S4 Fig — Northern analysis of total RNA isolated from the ssd1-d2 (W303-1A and UMY4432), ssd1-d2 elp3Δ (UMY3269 and UMY4439), SSD1 (UMY3385 and BY4741), and SSD1 elp3Δ (UMY4456 and MJY1036) strains grown in SC medium at 30°C. The blot was probed for tRNAUUULys, tRNAUUGGln, tRNAiMet, and 5.8S rRNA using radiolabeled oligonucleotides. (PDF) [file pgen.1008117.s004.pdf]

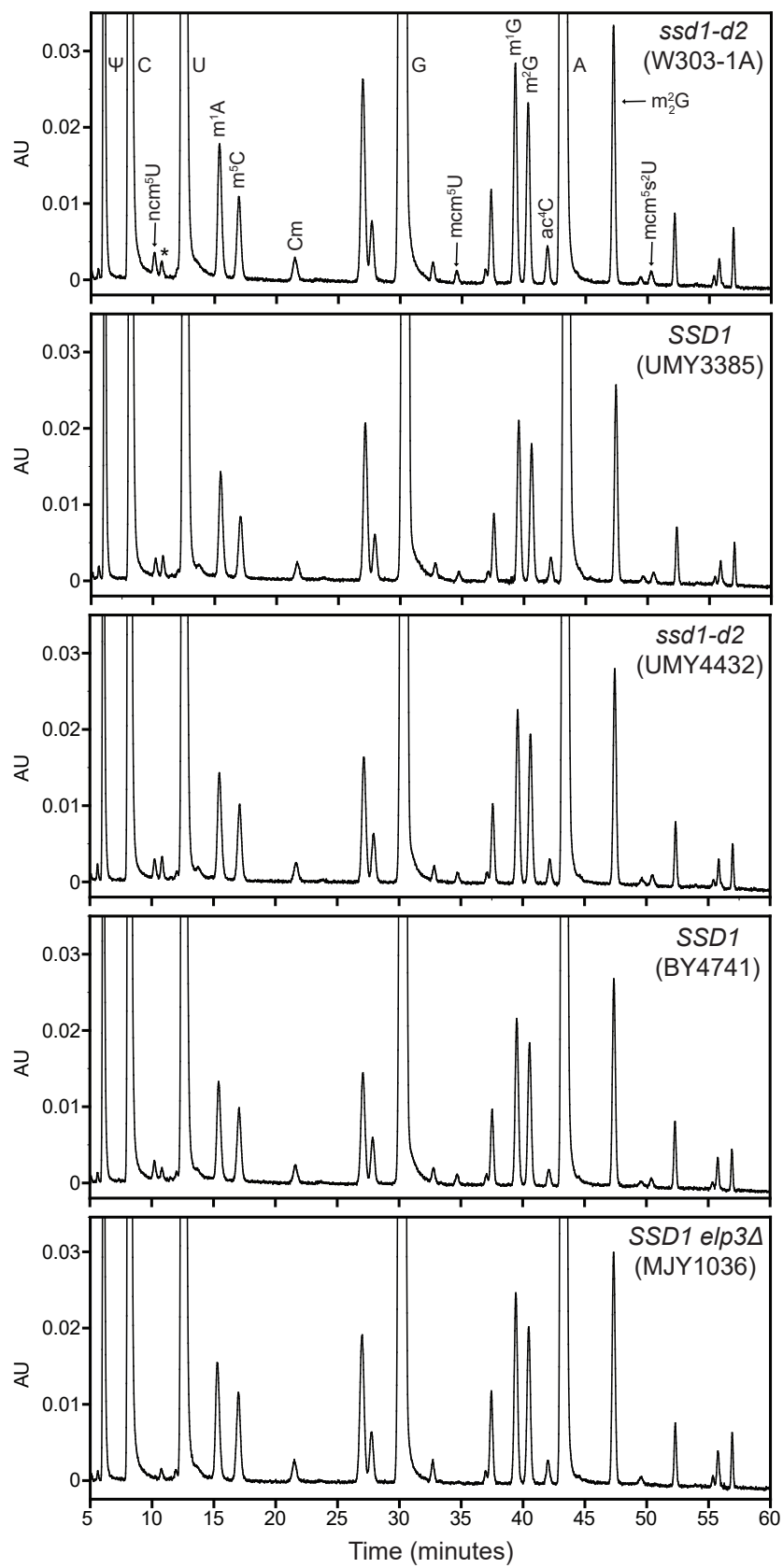

Supplement: S5 Fig — The peaks representing ncm5U, mcm5U, mcm5s2U, pseudouridine (Ψ), cytidine (C), uridine (U), guanosine (G), adenosine (A), 1-methyladenosine (m1A), 5-methylcytidine (m5C), 2'-O-methylcytidine (Cm), 1-methylguanosine (m1G), N2-methylguanosine (m2G), N4-acetylcytidine (ac4C), and N2, N2-dimethylguanosine (m22G) are indicated. The asterisk indicates a peak that is a contamination from the bacterial alkaline phosphatase. (PDF) [file pgen.1008117.s005.pdf]

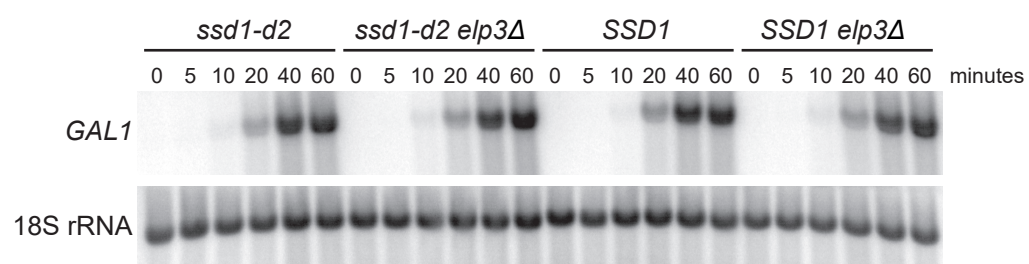

Supplement: S6 Fig — Northern analysis of total RNA isolated from the ssd1-d2 (W303-1A), ssd1-d2 elp3Δ (UMY3269), SSD1 (UMY3385) and SSD1 elp3Δ (UMY4456) strains. Cells were grown in SC medium containing 2% raffinose followed by induction of GAL1 transcription by the addition of 0.1 volumes 20% galactose. Time points after the addition of galactose are indicated above the lanes. The blot was probed for GAL1 transcripts using a randomly labelled DNA fragment. 18S rRNA was detected using a oligonucleotide probe. The blot is a representative of two independent experiments. (PDF) [file pgen.1008117.s006.pdf]

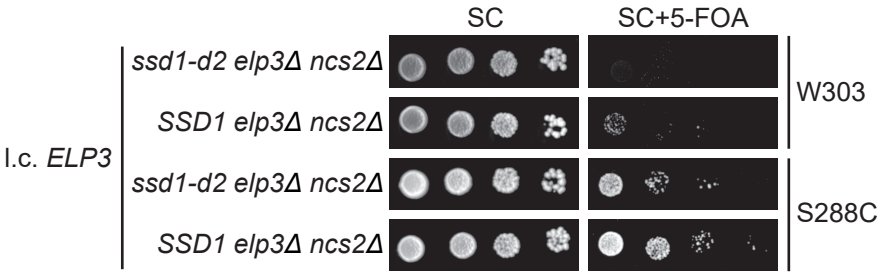

Supplement: S7 Fig — The figure shows a shorter incubation (2 days) of the plates in Fig 4A. (PDF) [file pgen.1008117.s007.pdf]
