# Supplementary material for: SSD1 suppresses phenotypes induced by the lack of Elongator-dependent tRNA modifications
Source: PLoS Genet. 2019 Aug 29;15(8):e1008117. doi: 10.1371/journal.pgen.1008117 (PMC6738719; doi:10.1371/journal.pgen.1008117)
Supplement: S1 Table — (DOCX) [file pgen.1008117.s008.docx]

S1 Table. Steady-state tRNA levels in *elp3Δ* cells carrying the indicated plasmids.

|  | Relative tRNA level^a^ | | | | | | | |
| --- | --- | --- | --- | --- | --- | --- | --- | --- |
| Plasmid | $\text{tRNA}_{\text{UUU}}^{\text{Lys}}$ | |  | $\text{tRNA}_{\text{UUG}}^{\text{Gln}}$ | |  | $\text{tRNA}_{\text{i}}^{\text{Met}}$ | |
|  | 30°C | 37°C |  | 30°C | 37°C |  | 30°C | 37°C |
| h.c. vector | 1.00 | 1.00 |  | 1.00 | 1.00 |  | 1.00 | 1.00 |
| l.c. *ELP3* | 1.35 ± 0.07 | 1.57 ±0.67 |  | 0.99 ± 0.05 | 1.06 ± 0.30 |  | 0.93 ± 0.04 | 1.25 ± 0.61 |
| h.c. *tK(UUU)*-*tQ(UUG)* | 2.00 ± 0.25 | 2.65 ± 0.85 |  | 2.07 ± 0.23 | 2.82 ± 0.62 |  | 0.96 ± 0.12 | 1.16 ± 0.47 |
| h.c. *PKC1* | 0.89 ± 0.21 | 1.54 ± 0.55 |  | 0.90 ± 0.21 | 1.32 ± 0.32 |  | 0.91 ± 0.08 | 1.35 ± 0.44 |

^a^ The signal for the indicated tRNA species was normalized to the corresponding 5.8S rRNA signal and the value expressed relative to that for the strain carrying the empty h.c. vector. The values represent the average from the blot shown in Fig 1B and two additional independent experiments. The standard deviation is indicated.
