# Supplementary material for: SSD1 suppresses phenotypes induced by the lack of Elongator-dependent tRNA modifications
Source: PLoS Genet. 2019 Aug 29;15(8):e1008117. doi: 10.1371/journal.pgen.1008117 (PMC6738719; doi:10.1371/journal.pgen.1008117)
Supplement: S2 Table — (DOCX) [file pgen.1008117.s009.docx]

**S2 Table.** Generation times of indicated strains grown at 30°C or 37°C.

| Background | Strain | Generation time (h)^a^ | |
| --- | --- | --- | --- |
|  |  | 30°C | 37°C |
| W303 | *ssd1-d2* (W303-1A) | 1.67 ± 0.10 | 1.96 ± 0.13 |
|  | *SSD1* (UMY3385) | 1.61 ± 0.12 | 1.84 ± 0.06 |
|  | *ssd1-d2 elp3Δ* (UMY3269) | 2.32 ± 0.20 | 3.53 ± 0.21 |
|  | *SSD1 elp3Δ* (UMY4456) | 1.99 ± 0.07 | 2.69 ± 0.21 |
| S288C | *ssd1-d2* (UMY4432) | 1.47 ± 0.02 | 1.91 ± 0.16 |
|  | *SSD1* (BY4741) | 1.43 ± 0.03 | 1.61 ± 0.04 |
|  | *ssd1-d2 elp3Δ* (UMY4439) | 2.26 ± 0.11 | 3.66 ± 0.22 |
|  | *SSD1 elp3Δ* (MJY1036) | 2.11 ± 0.04 | 2.68 ± 0.12 |

^a^ Growth rates were determined in SC medium. The values represent the average from four independent experiments and their standard deviations.
