# Supplementary material for: SSD1 suppresses phenotypes induced by the lack of Elongator-dependent tRNA modifications
Source: PLoS Genet. 2019 Aug 29;15(8):e1008117. doi: 10.1371/journal.pgen.1008117 (PMC6738719; doi:10.1371/journal.pgen.1008117)
Supplement: S3 Table — (DOCX) [file pgen.1008117.s010.docx]

S3 Table. Steady-state tRNA levels in the indicated strains.

| Background | Strain | Relative tRNA level^a^ | | | |
| --- | --- | --- | --- | --- | --- |
|  |  | $\text{tRNA}_{\text{UUU}}^{\text{Lys}}$ |  | $\text{tRNA}_{\text{UUG}}^{\text{Gln}}$ | $\text{tRNA}_{\text{i}}^{\text{Met}}$ |
| W303 | *ssd1-d2* (W303-1A) | 1.00 |  | 1.00 | 1.00 |
|  | *ssd1-d2 elp3Δ* (UMY3269) | 0.88 ± 0.14 |  | 1.01 ± 0.07 | 0.90 ± 0.07 |
|  | *SSD1* (UMY3385) | 1.23 ± 0.20 |  | 1.15 ± 0.04 | 1.09 ± 0.19 |
|  | *SSD1 elp3Δ* (UMY4456) | 1.09 ± 0.12 |  | 1.20 ± 0.22 | 1.02 ± 0.04 |
| S288C | *ssd1-d2* (UMY4432) | 1.00 |  | 1.00 | 1.00 |
|  | *ssd1-d2 elp3Δ* (UMY4439) | 0.81 ± 0.06 |  | 0.98 ± 0.10 | 0.97 ± 0.01 |
|  | *SSD1* (BY4741) | 1.09 ± 0.08 |  | 1.09 ± 0.09 | 1.06 ± 0.03 |
|  | *SSD1 elp3Δ* (MJY1036) | 1.06 ± 0.05 |  | 0.99 ± 0.06 | 0.98 ± 0.09 |

^a^ The signal for the indicated tRNA species was normalized to the corresponding 5.8S signal and the value expressed relative to that for the respective *ssd1-d2* strain, which was set to 1*.* The values represent the average from the blot shown in S4 Fig and two additional independent experiments. The standard deviation is indicated.
