# Supplementary material for: SSD1 suppresses phenotypes induced by the lack of Elongator-dependent tRNA modifications
Source: PLoS Genet. 2019 Aug 29;15(8):e1008117. doi: 10.1371/journal.pgen.1008117 (PMC6738719; doi:10.1371/journal.pgen.1008117)
Supplement: S4 Table — (DOCX) [file pgen.1008117.s011.docx]

S4 Table. Relative amounts of selected modified nucleosides in total tRNA from various strains.

| Background | Strain | Modified nucleoside^a^ | | | | | | | |
| --- | --- | --- | --- | --- | --- | --- | --- | --- | --- |
|  |  | ncm^5^U | mcm^5^U | mcm^5^s^2^U | m^1^A | m^5^C | m^1^G | m^2^G | ac^4^C |
| W303 | *ssd1-d2* (W303-1A) | 1.00 | 1.00 | 1.00 | 1.00 | 1.00 | 1.00 | 1.00 | 1.00 |
|  | *SSD1* (UMY3385) | 0.95 ± 0.08 | 1.00 ± 0.21 | 0.87 ± 0.13 | 1.03 ± 0.02 | 1.14 ± 0.02 | 1.03 ± 0.01 | 0.94 ± 0.08 | 1.07 ± 0.12 |
|  | *ssd1-d2 elp3Δ* (UMY3269) | -^b^ | - | - | 1.01 ± 0.02 | 1.00 ± 0.04 | 0.97 ± 0.02 | 1.01 ± 0.03 | 0.89 ± 0.10 |
|  | *SSD1 elp3Δ* (UMY4456) | - | - | - | 1.03 ± 0.06 | 1.14 ± 0.02 | 0.99 ± 0.03 | 1.01 ± 0.04 | 0.93 ± 0.22 |
| S288C | *ssd1-d2* (UMY4432) | 0.99 ± 0.07 | 1.18 ± 0.16 | 0.86 ± 0.11 | 1.03 ± 0.05 | 1.14 ± 0.08 | 0.97 ± 0.03 | 1.04 ± 0.04 | 1.09 ± 0.20 |
|  | *SSD1* (BY4741) | 0.97 ± 0.08 | 1.01 ± 0.22 | 0.83 ± 0.17 | 1.02 ± 0.06 | 1.16 ± 0.04 | 0.98 ± 0.01 | 1.03 ± 0.06 | 0.97 ± 0.26 |
|  | *ssd1-d2 elp3Δ* (UMY4439) | - | - | - | 0.99 ± 0.07 | 1.16 ± 0.03 | 0.97 ± 0.03 | 1.04 ± 0.04 | 0.86 ± 0.39 |
|  | *SSD1 elp3Δ* (MJY1036) | - | - | - | 1.08 ± 0.07 | 1.19 ± 0.03 | 1.00 ± 0.02 | 1.05 ± 0.05 | 1.16 ± 0.34 |

^a^ The peaks for ncm^5^U, mcm^5^U, mcm^5^s^2^U, m^1^A (1-methyladenosine), m^5^C (5-methylcytidine), m^1^G (1-methylguanosine), m^2^G (*N*^2^-methylguanosine), and ac^4^C (*N*^4^-acetylcytidine) were integrated and the values were normalized to the value for pseudouridine (Ψ). The normalized values were expressed relative to the corresponding values from the W303-1A strain, which was set to 1. The values represent the average of three independent experiments and their standard deviations.

^b^ Below the detection limit.
