# Supplementary material for: SSD1 suppresses phenotypes induced by the lack of Elongator-dependent tRNA modifications
Source: PLoS Genet. 2019 Aug 29;15(8):e1008117. doi: 10.1371/journal.pgen.1008117 (PMC6738719; doi:10.1371/journal.pgen.1008117)
Supplement: S5 Table — (DOCX) [file pgen.1008117.s012.docx]

S5 Table. Yeast strains used in this study.

| Strain | Genotype | Source or reference |
| --- | --- | --- |
| BY4741 | *MATa his3Δ1 leu2Δ0 met15Δ0 ura3Δ0 SSD1* | [67] |
| BY4742 | *MATα his3Δ1 leu2Δ0 lys2Δ0 ura3Δ0 SSD1* | [67] |
| UMY2836 | Diploid between BY4741 and BY4742 | This lab |
| MJY1021 | *MATα his3Δ1 leu2Δ0 lys2Δ0 ura3Δ0 SSD1 ncs2::kanMX4* | This study |
| MJY1036 | *MATa his3Δ1 leu2Δ0 met15Δ0 ura3Δ0 SSD1 elp3::kanMX4* | This study |
| MJY1037 | *MATα his3Δ1 leu2Δ0 lys2Δ0 ura3Δ0 SSD1 elp3::kanMX4* | This study |
| MJY1058 | *MATa his3Δ1 leu2Δ0 met15Δ0 ura3Δ0 SSD1 elp3::kanMX4 ncs2::kanMX4* | This study |
| MJY1159 | *MATa his3Δ1 leu2Δ0 met15Δ0 ura3Δ0 ssd1-d2 elp3::KanMX4 ncs2::kanMX4* pRS316*-ELP3* | This study |
| UMY4449 | *MATa his3Δ1 leu2Δ0 met15Δ0 ura3Δ0 ssd1-d2 elp3::kanMX4 ncs2::kanMX4* | This study |
| UMY4432 | *MATa his3Δ1 leu2Δ0 met15Δ0 ura3Δ0 ssd1-d2* | This study |
| UMY4433 | *MATα his3Δ1 leu2Δ0 lys2Δ0 ura3Δ0 ssd1-d2* | This study |
| UMY4559 | *MATa his3Δ1 leu2Δ0 met15Δ0 ura3Δ0 ssd1::kanMX4* | This study |
| MJY1227 | *MATα his3Δ1 leu2Δ0 met15Δ0 lys2Δ0 ura3Δ0 ssd1::kanMX4 elp3::kanMX4* | This study |
| UMY4434 | Diploid between UMY4432 and UMY4433 | This study |
| UMY4438 | *MATα his3Δ1 leu2Δ0 ura3Δ0 ssd1-d2 elp3::kanMX4* | This study |
| UMY4439 | *MATa his3Δ1 leu2Δ0 met15Δ0 ura3Δ0 ssd1-d2 elp3::kanMX4* | This study |
| UMY4442 | *MATα his3Δ1 leu2Δ0 lys2Δ0 ura3Δ0 ssd1-d2 ncs2::kanMX4* | This study |
| W303-1A | *MATa leu2-3,112 trp1-1 can1-100 ura3-1 ade2-1 his3-11,15 ssd1-d2* | [69] |
| W303-1B | *MATα leu2-3,112 trp1-1 can1-100 ura3-1 ade2-1 his3-11,15 ssd1-d2* | [69] |
| UMY3269 | *MATa leu2-3,112 trp1-1 can1-100 ura3-1 ade2-1 his3-11,15 ssd1-d2 elp3::kanMX4* | [21] |
| UMY2843 | *MATα leu2-3,112 trp1-1 can1-100 ura3-1 ade2-1 his3-11,15 ssd1-d2 elp3::kanMX4* | [74] |
| UMY3442 | *MATα leu2-3,112 trp1-1 can1-100 ura3-1 ade2-1 his3-11,15 ssd1-d2 ncs2::kanMX4* | [20] |
| UMY3385 | *MATa leu2-3,112 trp1-1 can1-100 ura3-1 ade2-1 his3-11,15 SSD1* | [43] |
| UMY3386 | *MATα leu2-3,112 trp1-1 can1-100 ura3-1 ade2-1 his3-11,15 SSD1* | [43] |
| UMY3387 | Diploid between UMY3385 and UMY3386 | [43] |
| MJY1019 | *MATα leu2-3,112 trp1-1 can1-100 ura3-1 ade2-1 his3-11,15 SSD1 ncs2::kanMX4* | This study |
| UMY4456 | *MATa leu2-3,112 trp1-1 can1-100 ura3-1 ade2-1 his3-11,15 SSD1 elp3::kanMX4* | This study |
| UMY4457 | *MATα leu2-3,112 trp1-1 can1-100 ura3-1 ade2-1 his3-11,15 SSD1 elp3::kanMX4* | This study |
| UMY4558 | *MATa leu2-3,112 trp1-1 can1-100 ura3-1 ade2-1 his3-11,15 ssd1::kanMX4* | This study |
| UMY4574 | *MATa leu2-3,112 trp1-1 can1-100 ura3-1 ade2-1 his3-11,15 ssd1::kanMX4 elp3::kanMX4* | This study |
| UMY4467 | *MATa leu2-3,112 trp1-1 can1-100 ura3-1 ade2-1 his3-11,15 SSD1 elp3::kanMX4 ncs2::kanMX4* | This study |
| UMY4454 | *MATa leu2-3,112 trp1-1 can1-100 ura3-1 ade2-1 his3-11,15 ssd1-d2 elp3::kanMX4 ncs2::kanMX4 pRS316-ELP3* | This study |
| UMY2584 | *MATa leu2-3,112 trp1-1 can1-100 ura3-1 ade2-1 his3-11,15 ssd1-d2 TELVIIL::URA3 TELVR::ADE2* | [21] |
| UMY3790 | *MATa leu2-3,112 trp1-1 can1-100 ura3-1 ade2-1 his3-11,15 ssd1-d2 TELVIIL::URA3 TELVR::ADE2 elp3::kanMX4* | [21] |
