# Supplementary material for: SSD1 suppresses phenotypes induced by the lack of Elongator-dependent tRNA modifications
Source: PLoS Genet. 2019 Aug 29;15(8):e1008117. doi: 10.1371/journal.pgen.1008117 (PMC6738719; doi:10.1371/journal.pgen.1008117)
Supplement: S6 Table — (DOCX) [file pgen.1008117.s013.docx]

S6 Table. Plasmids used in this study

| Plasmid | Description | Source or reference |
| --- | --- | --- |
| pRS316 | YCp, *URA3* | [68] |
| pRS315 | YCp, *LEU2* | [68] |
| pRS425 | YEp, *LEU2* | [70] |
| pABY2160 | pRS425-*MID2* | This study |
| pMJ120 | pRS425-*WSC2* | This study |
| pABY2176 | pRS425-*ROM1* | This study |
| pABY2128 | pRS425-*RHO1* | This study |
| pABY2177 | pRS315-*RHO1* | This study |
| pABY2129 | pRS425-*PKC1* | This study |
| pABY2130 | pRS425-*BCK1* | This study |
| pABY2239 | pRS315-*BCK1* | This study |
| pABY2131 | pRS425-*MPK1* | This study |
| pABY2240 | pRS315-*MPK1* | This study |
| pABY2227 | pRS315-*SSD1* | This study |
| pABY2139 | Frameshift construct (CUU AAA C) | [52] |
| pABY2144 | In-frame control | [52] |
| pABY1707 | pRS425-*tK(UUU)-tQ(UUG)* | [74] |
| pABY1514 | pRS315-*ELP3* | [5] |
| pABY1481 | pRS316-*ELP3* | [40] |
