# Supplementary material for: SSD1 suppresses phenotypes induced by the lack of Elongator-dependent tRNA modifications
Source: PLoS Genet. 2019 Aug 29;15(8):e1008117. doi: 10.1371/journal.pgen.1008117 (PMC6738719; doi:10.1371/journal.pgen.1008117)
Supplement: S7 Table — (DOCX) [file pgen.1008117.s014.docx]

S7 Table. Oligonucleotides used in this study

| Name | Purpose | Sequence | Restriction site |
| --- | --- | --- | --- |
| o3258 | *MID2* cloning | AAAAACTAGTTTGCTTTCATAATCTGCAAAT | *Spe*I |
| o3259 | *MID2* cloning | AAAACCCGGGCAGTGGAACGTTAAAGCACT | *Sma*I |
| o2945 | *Wsc2* cloning | AAAAGCGGCCGCTTGTGATCTAGCACTTCTC | *Not*I |
| o2946 | *Wsc2* cloning | AAAAGTCGACGTAATGTGGAGATCATCG | *Sal*I |
| o3248 | *ROM1* cloning | AAAAACTAGTACTTTTGCCATCTTATACTCATC | *Spe*I |
| o3249 | *ROM1* cloning | AAAACCCGGGCTTCAATACGGTCAGAATTATC | *Sma*I |
| o3106 | *RHO1* cloning | AAAAGCGGCCGCAGGTTGGGTTATGGAACCTT | *Not*I |
| o3107 | *RHO1* cloning | AAAAGTCGACTTGCCAGGTGTTAAGAAGG | *Sal*I |
| o3104 | *PKC1* cloning | AAAAGCGGCCGCCGCGAACTCGTAAGTAGAAAA | *Not*I |
| o3105 | *PKC1* cloning | AAAAGTCGACACATTCTTCAAATGCCTGC | *Sal*I |
| o3118 | *Bck1* cloning | AAAACCCGGGTCCATATTTGGTGACCGA | *Sma*I |
| o3119 | *Bck1* cloning | AAAAGAGCTCGAGGACCTTCCTGATGAAAG | *Sac*I |
| o3102 | *MPK1* cloning | AAAAGCGGCCGCGAGCGGTAACTATGGACACC | *Not*I |
| o3103 | *MPK1* cloning | AAAAGTCGACGAGTACGATTAAGATAAGCGTCG | *Sal*I |
| o2975 | *SSD1* cloning; *ssd1::kanMX4* amplification | AAAAGGATCCTCACGAGTATTTTCGCTC | *Bam*HI |
| o2976 | *SSD1* cloning; *ssd1::kanMX4* amplification | AAAAGAGCTCCGGAAAAATTACCCAGC | *Sac*I |
| o3465 | *ssd1Δ* confirmation | TGTCATTTGTTTAGTTCAAGG |  |
| o3466 | *ssd1Δ* confirmation | GCTACAGATTAAACGCTGAC |  |
| o797 | *elp3::kanMX4* amplification | GCTTACACTTCGTTCCTTCC |  |
| o798 | *elp3::kanMX4* amplification | CAGTGAGAGAAGGAGAAAGC |  |
| o800 | *elp3Δ* confirmation | CATGTACGGTCGCTTGAGGT |  |
| o799 | *elp3Δ* confirmation | CGTGCAATTGACCGAACGTG |  |
| o3472 | *ssd1::URA3* amplification | CGTTGGCCAATCACATCTTTGCATCCATTTGGTATTTTAGTGATGACGGTGAAAACCTCT |  |
| o3473 | *ssd1::URA3* amplification | AACCGACAGCGTGGCTGATTCCTTGCCAGGGGCCAACGACCGGCCTATTGGTTAAAAAATG |  |
| o3469 | *ssd1-d2* amplification | ATGTCGTTGCTGTTTTGGAC |  |
| o3478 | *ssd1-d2* amplification | CCTAAAGTTCTATCCAGGATG |  |
| o1423 | *ncs2::kanMX4* amplification | GATCTTTTCCACTGGTCGTC |  |
| o1424 | *ncs2::kanMX4* amplification | CTACTTAAAGCCCAAGCCTC |  |
| o1991 | *ncs2Δ*  confirmation | tggtatcggtctgcgattcc |  |
| o1425 | *ncs2Δ* confirmation | CTACGTCGACGGTGAGTGGTGGAGTTCCTC |  |
| o1128 | $\text{tRNA}_{\text{UUU}}^{\text{Lys}}\text{ }$probe | CCCTGACATTTCGGTTAA |  |
| o1439 | $\text{tRNA}_{\text{UUG}}^{\text{Gln}}$ probe | CCACTACACTATAGGACC |  |
| oMJ561 | $\text{tRNA}_{\text{i}}^{\text{Met}}\text{ }$ probe | GGACATCAGGGTTATGAGCC |  |
| o591 | 5.8S rRNA probe | GCGTTGTTCATCGAT |  |
